# Assessment of nicotine dependence among tobacco users visiting outreach programs in Dharan, Nepal: a cross-sectional study

**DOI:** 10.1186/s12889-021-11535-9

**Published:** 2021-08-06

**Authors:** Krishna Subedi, Ashish Shrestha, Tarakant Bhagat

**Affiliations:** 1 Dental Department, Pokhara Academy of Health Sciences, Pokhara, Nepal; 2 Department of Public Health Dentistry, CODS, B.P Koirala Institute of Health Sciences, Dharan, Nepal

**Keywords:** FTND, Nicotine dependence, Smokeless tobacco, Smokers

## Abstract

**Background:**

Nicotine is a highly addictive substance present in tobacco. This study was conducted to assess the level of nicotine dependence among smokers and smokeless tobacco users visiting dental outreach programs of B.P. Koirala Institute of Health Sciences -Dharan, Nepal.

**Methods:**

A cross sectional study was conducted from June 2018 to April 2019. A total of 726 people were selected from participants of dental outreach programs of 6 districts using convenience sampling technique. The data collection was done using semi-structured questionnaire through face-to-face interview by a single researcher. History of tobacco use and level of nicotine dependency was measured using Nepali translated and validated form of Fagerström Test for Nicotine Dependence for smoking and smokeless tobacco. The mean age of the tobacco users was 39.55 ± 15.57. Descriptive statistics including the mean, median, percentage, standard deviations and interquartile range were computed. Chi-square test, Fisher’s exact test, univariate and bivariate logistic regression were used where needed.

**Results:**

Nicotine dependence (moderate and severe) was found in 80% of smokeless tobacco users and 48% of smokers. Among the smokeless tobacco users, nicotine dependency was found to be more with increase in duration of tobacco use (AOR = 50.25, 95%CI = 3.51–718.62, *p* = 0.004), low socioeconomic status (AOR = 6.27, 95%CI = 1.30–30.31, *p* = 0.02), less number of tobacco packets used per day and tried to quit tobacco use in last 1 year. Among smokers nicotine dependency was found to be significantly higher with smoking more than 10 cigarettes per day (AOR = 7.14, 95% CI = 2.00–25.40, *p* = 0.002).

**Conclusions:**

The study concluded that level of nicotine dependence for both smoking and smokeless tobacco was high among the people visiting dental outreach programs. It is high time to develop a policy to control tobacco use along with creating tobacco cessation centers. Currently, tobacco control program is mostly focusing on smoking. However, it is also important to incorporate smokeless tobacco control at policy level.

## Background

Tobacco use causes premature death and disease worldwide which is mostly preventable [1]. The world has faced tobacco as an epidemic and is considered as one of the major public health threats to the entire humankind killing more than 8 million people in a year. Most of these deaths are the direct result of tobacco use, 7 million resulting from first hand and 1.2 million from second-hand smoking. There are more than 1 billion smokers residing worldwide, out of which around 80% are living in middle-and low-income countries (LICs) like India, Bangladesh and Nepal. Tobacco related illness and death is heaviest in these regions as compared to other regions of the world [2]. Tobacco use is considered as a severe public health threat in the South-East Asia (SEA) Region. This region has almost one quarter of all smokers in the world [3]. More than 300 million people around the world use smokeless tobacco (ST) products and its harmful nature make ST consumption a global public health issue. The highest rate of ST consumption is in SEA region [4].

International Classification of Disease (ICD-10) considered tobacco dependence itself as a chronic disease [5]. Nicotine is a highly addictive substance, which is present in tobacco along with other 4000 chemicals [2]. Constant use of tobacco products leads to addiction among high proportion of users [6]. Nicotine causes addiction by acting on nicotinic cholinergic receptors which triggers the release of neurotransmitters that produce psychoactive effects that are rewarding [7]. One person prematurely dies in every six seconds due to addiction of tobacco. One in two long-term smokers mostly in low- and middle-income countries will die from tobacco addiction. This epidemic reflects the highly addictive nature of tobacco, and specifically of nicotine [8].

There is a decreasing trend of nicotine use in most of the high-income countries whereas increasing trend in most of the low and lower-middle income countries [9]. Nicotine dependence is regarded as a primary problem, which is worthy of ongoing attention from every clinician. Complications of nicotine dependence consist of diseases caused by tobacco use or conditions worsened by it [6]. Addiction of tobacco is found to be parallel as of heroin and cocaine [10]. Quitting tobacco is not easy, as tobacco dependence is a cluster of physical, behavioral, and psychological or emotional phenomena [11].

More than 21,000 children (10–14 years old) and 30,46,000 adults (15+ years old) continue to use tobacco daily in Nepal. More than 27,100 people die each year in Nepal due to tobacco related disease [12]. A cross sectional study done in Nepal showed that nicotine dependence among smokers was found to be 20.4% and 30.3% as high and moderate respectively [13]. Various studies have been done related to tobacco in Nepal but there is dearth of studies regarding nicotine dependence among smoking and smokeless tobacco users. Therefore, this study was done with the objective to assess the level of nicotine dependence among smokers and smokeless tobacco users visiting outreach programs of the Department of Public Health Dentistry, BPKIHS-Dharan, Nepal.

## Methods

### Study design

A questionnaire based cross sectional study was conducted from June 2018 to April 2019 among people aged more than 18 years who visited outreach programs of the Department of Public Health Dentistry, CODS, BPKIHS - Dharan, Nepal including dental camps. This study adhered to STROBE guidelines. BPKIHS is an autonomous Health Sciences University which is located at eastern part of Nepal in province one. It is the only Government University with tertiary care in eastern part of Nepal. It has extended its continued health services through teaching district concept to primary health care centers, district hospitals and zonal Hospitals in different parts of the region. Study was conducted during regular outreach programs of Department of Public Health Dentistry including dental camps.

### Setting and participants of the study

Data collection was done in five districts out of 14 districts in state number one and one district from state number two using convenience sampling technique. Outreach programs were conducted once a week in one of the centers. Dental camps were organized once a month in support with local authorities for the needy people. All the people who had fulfilled the eligibility criteria and had given written informed consent were included in this study. Outreach programs of the department of public health dentistry included two primary health care center, one family health care center, two health post and the dental camps in 13 various sites. Out of these centers three were located in urban areas, two were located in rural areas. Dental camps meanwhile were organized in rural areas.

### Eligibility criteria

People aged more than 18 years who had visited outreach programs of the Department of Public Health Dentistry, BPKIHS-Dharan, Nepal including dental camps were included in this study. People with medical or psychiatric condition who were unable to respond to the questions were excluded.

### Ethical approval

Ethical approval was obtained from the Institutional Review Committee, B.P.Koirala Institute of Health Sciences, Dharan, Nepal (Ref. No: 398/074/075-IRC and Code No: IRC/1191/017). Signed informed consent was obtained from each participant.

### Study size

The sample size was calculated by taking the prevalence of tobacco use in Nepal (30.8%) [13]; considering 95% CI, 5% absolute precision and 15% non-response rate, calculated sample size was 378. Sample size was calculated using following formula [(Z_α/2_)^2^pq/L^2^]. As convenience method of sampling was used, the number from each center was not predetermined. Sample collection was done till the sample size was reached.

### Demographic characteristics

Demographic variables consisting of age, sex, occupation, marital status, education (illiterate, primary level, middle class level, secondary level, high school level and graduate or postgraduate) income of the family and socioeconomic status (SES) was calculated using Kuppuswamy scale and classified as per the modifications done in the year 2009 (Ghosh and Ghosh, 2009) using current consumer price index for the year 2017. The current consumer price index was obtained online from *Nepal Rastra Bank* website (Nepal RB 2017) and the conversion factor was calculated (Conversion factor = Consumer Price Index 2017 divided by Consumer Price Index of 1976). The computed conversion factor was 26.7 (114.8/4.3). For simplicity, SES was categorized into upper (26–29), middle (11–25) and lower (≤5–10) class.

Other variables used in this study were:

1. Do you drink alcohol? (yes or no).
2. Do any other members in your family smoke/tobacco chew? (yes or no).
3. At what age do you started smoking/chewing tobacco?
4. Total duration of smoking/tobacco chewing in years.
5. Ever tried to quit smoking/tobacco chewing in last 1 year: yes/no.

The following forms of tobacco use were recorded for tobacco prevalence. **Smoking**: manufactured cigarettes, cigar, bidis and pipe. **Tobacco chewing**: khaini, gutka, loose-leaf of tobacco, and gul.

### Nicotine dependence

A standard questionnaire proforma of Fagerstrom Test for Nicotine Dependence (FTND Revised Version) for smoking given by Heatherton et al. [14] and smokeless form of tobacco given by Ebbert et al. [15] was translated and validated in Nepali language through standard WHO guidelines. During back translation, the meanings of some questions were modified, so the questions were modified keeping the same scoring criteria (Appendix 1). This modified standard translated and validated questionnaire in Nepali language was administered to each participant by face-to-face interview by the single investigator. Each Smoking and smokeless tobacco questionnaire consisted of 6 questions, carrying score/point based on the answer given. The score ranged from 0 to 10. Based on the calculated score dependency was categorized as highly dependent (7–10), moderately dependent (4–6) and minimally dependent (< 4). Further it was categorized into nicotine dependency (highly and moderately dependent) and no nicotine dependency (minimally dependent).

The higher the Fagerström score, the more intense was the patient’s physical dependence on nicotine. Higher scores indicated that treatment of withdrawal symptoms, usually with nicotine replacement therapy, will be an important factor in the patient’s plan of care**.**

#### Operational definitions

**a. Current smokers**: People who reported smoking at least 100 cigarettes during their lifetime and who, at the time they participated in a survey, reported smoking every day or some days. **Current smokeless tobacco product users:** Chewing tobacco at least once during their lifetime and, at the time of the survey, using every day or some days. **b**. **Nontobacco users:** Never used tobacco. **c. Ex-tobacco user:** Stopped more than 1 month prior to the examination.

All the subjects were advised for smoking/tobacco cessation and tobacco cessation counselling was given to every one after administration of questionnaire. Behavioral cognitive therapy was given involving 5 “A” and 5 “R” technique.

### Statistical analysis

After completion of the survey, data obtained was entered in Microsoft Excel Sheet version 2007 and analyzed using the Statistical Package for Social Sciences (SPSS version 11.5). Descriptive statistics including the mean, median, percentage, standard deviations and interquartile range were computed. FTND score was categorized as high, moderate and minimal dependent. Further it was categorized as nicotine dependent (high and moderate) and nicotine not dependent (minimal dependent) for analysis of association. Chi-square test and Fisher’s exact tests were used to compare the proportion difference between categorical variables where needed. Relationship between FTND score and different study variables (gender, mean age of initiation and total duration of tobacco use, SES, marital status, occupation, alcohol use, number of cigarettes smoked per day, number of packets/cans of smokeless tobacco chewed/day and family member smoking/chewing tobacco) were computed for odds ratio. Univariate and binary logistic regression analyses were performed to find the association between the different variables and FTND score and nicotine dependence and their odds ratios (OR) were calculated.

## Results

A total of 13 dental camps were organized during this study period. Out of 726 (406 males and 320 females) people surveyed in the study, 238 (32.8%) were tobacco users. The prevalence of tobacco use in any form for males and females was found to be 42.8% (174) and 20.0% (64) respectively (Table 6 in Appendix 2). The age ranged from 18 to 82 years with mean age and standard deviation (S.D) of 39.55 ± 15.57 (male 36.12 ± 15.40 and female 48.89 ± 11.84). More number of married people used tobacco (74.8%). Majority of people (65.1%) had education of school leaving certificate or lower. Only 6.3% had graduate/postgraduate level of education. Female had low level of education as compared to male (*p* = < 0.001). The majority of the people belonged to middle socioeconomic class followed by lower socioeconomic class (Table 1).

**Table 1:** Socio-demographic characteristics of the tobacco users visiting outreach program of Dharan, Nepal in June 2018 to April 2019 (*n* = 238)

Socio-demographic and general profile	Male ***n*** = 174 (73.11%)	Female ***n*** = 64 (26.89%)	Total *n* = 238 (100.0%)	***P*** value
**Age in years**				**χ2 = 96.76**
≤ 29	77 (44.3%)	5 (7.8%)	82 (34.5%)	***P*** **= 0.0001**
30–39	36 (20.7%)	10 (15.6%)	46 (19.3%)	
40–49	22 (12.6%)	17 (26.6%)	39 (16.4%)	
50–59	23 (13.2%)	20 (31.3%)	43 (18.1%)	
60	16 (9.2%)	12 (18.8%)	28 (11.8%)	
Mean age ± SD	36.12 ± 15.40	48.89 ± 11.84	39.55 ± 15.57	
**Marital Status**				**Fisher exact value = 27.45**
Unmarried	57 (32.8%)	0 (0.0%)	57 (24.0%)	***P*** **= 0.000**
Married	117 (67.2%)	64 (100.0%)	181 (76.0%)	
**Levels of Education**				**Fisher exact value = 87.92**
Illiterate	12 (6.9%)	30 (46.9%)	42 (17.6%)	***P*** **= 0.000**
Primary Level (≤class 5)	19 (10.9%)	16 (25.0%)	35 (14.7%)	
Middle (6–8)	21 (12.1%)	13 (20.3%)	34 (14.3%)	
Secondary Level (SLC)	42 (24.1%)	2 (3.1%)	44 (18.5%)	
Higher secondary Level (+ 2/IA)	66 (37.9%)	2 (3.1%)	68 (28.6%)	
Graduate/Postgraduate	14 (8.0%)	1 (1.6%)	15 (6.3%)	
**Occupation**				**Fisher exact value = 21.61**
Employed	22 (12.6%)	2 (3.1%)	24 (10.1%)	***P*** **= 0.000**
Agriculture/Shop	63 (36.2%)	36 (56.3%)	99 (41.6%)	
Skilled Worker	21 (12.1%)	0	21 (8.8%)	
Unskilled Worker	3 (1.7%)	0	3 (1.3%)	
Economically inactive	55 (31.6%)	25 (39.1%)	80 (33.6%)	
Unemployed	10 (5.7%)	1 (1.6%)	11 (4.6%)	
**Socio-Economic class**				**χ2 = 41.61**
Lower	41 (23.6%)	44 (68.8%)	85 (35.7%)	***P*** **< 0.001**
Middle	133 (76.4%)	20 (31.3%)	153 (64.3%)	

*P* < 0.05 statistically significant

Majority of the tobacco users (127, 53.4%) consumed alcohol. Males (102, 58.6%) consumed more alcohol as compared to females (25, 39.1%). Alcohol use was seen in more percentage of people who were using both form of tobacco (73.7% in males versus 42.8% in females) followed by smoking (64.8% in male versus 55.0% in female) and least by ST users (46.3% in male versus 29.7% in female). Among the tobacco users 44.5% (106) had one or more family members who also used tobacco.

The mean age of initiation of tobacco use for smokeless tobacco was 21.0 ± 8.8. However, it was earlier in case of smoking with mean age of initiation 19.1 ± 5.9. However, females initiated smokeless tobacco earlier than males (20.0 ± 6.8 vs 21.6 ± 9.6) but males started smoking at earlier age than females (18.9 ± 5.2 Vs 19.9 ± 8.4).

The median duration of tobacco chewing was 17 years (IQR: 6–31). The median duration of tobacco chewing for male and female was 10 years (IQR: 4.7–26) and 25 years (IQR: 14.2–37.7) respectively. The median duration of smoking was 11.5 (IQR: 4–31). The median duration of smoking for male and female was found to be 6 years (IQR: 4–26) and 38 years (IQR: 24–42) respectively.

Only 24.2% (64) tried to quit tobacco at least once or twice in the last 1 year. More percentage of male tobacco users (51, 26.4%) tried to quit in comparison to female (13, 18.3%).

Among smokers, 29.9% (*n* = 40) had smoked the first cigarette within 5 min whereas 32.8% (*n* = 44) had smoked after 60 min of waking up. Almost 31% of smokers felt difficult when they were not allowed to smoke in the smoking restricted areas (temple, library, cinema hall etc.). About 60% smokers felt difficult to quit first cigarette in the morning. Most of the smokers (82.8%) smoked ≤10 cigarettes per day. Only 12.7% of smokers smoked more frequently during the first hours after waking up than during the rest of the day. Majority of the female smokers (74.1%, *n* = 20, *P* = 0.04) smoked even if they were ill and bedridden most of the day as compared to males (Table 2).

**Table 2:** Percentage of males and females who responded to FTND categories (Smoking form) visiting outreach program of Dharan, Nepal in June 2018 to April 2019 (*n* = 134)

Questions	Answers	Male (***n*** = 107)	Female (***n*** = 27)	Total (n = 134)	***P*** value
1.when do you smoke your first cigarette after waking up?	Within 5 min	29 (27.1%)	11 (40.7%)	40 (29.9%)	**Fisher exact value = 2.80** ***P*** **= 0.42**
	6–30 min	20 (18.7%)	6 (22.2%)	26 (19.4%)	
	31–60 min	20 (18.7%)	4 (14.8%)	24 (17.9%)	
	After 60 min	38 (35.5%)	6 (22.2%)	44 (32.8%)	
2.Do you feel difficult when you are not allowed to smoke in the smoking restricted areas (like temple, library, Cinema hall etc.)	Yes	32 (29.9%)	9 (33.3%)	41 (30.6%)	**χ2 = 0.11** ***P*** **= 0.73**
	No	75 (70.1%)	18 (66.7%)	93 (69.4%)	
3.Which cigarette is most difficult for you to quit?	The first one in the morning	63 (58.9%)	18 (66.7%)	81 (60.4%)	**χ2 = 0.54** ***P*** **= 0.46**
	Any other	44 (41.1%)	9 (33.3%)	53 (39.6%)	
4. How many cigarettes do you smoke per day?	10 or less	90 (84.1%)	21 (77.8%)	111 (82.8%)	**Fisher exact value = 3.39** ***P*** **= 0.21**
	11–20	17 (15.9%)	5 (18.5%)	22 (16.4%)	
	21–30	0(%)	1 (3.7%)	1 (0.7%)	
5. Do you smoke more frequently during the first hours after waking than during the rest of the day?	Yes	11 (10.3%)	6 (22.2%)	17 (12.7%)	**χ2 = 2.77** ***P*** **= 0.09**
	No	96 (89.7%)	21 (77.8%)	117 (87.3%)	
6. Do you smoke even if you are ill and bedridden most of the day?	Yes	56 (52.3%)	20 (74.1%)	76 (56.7%)	**χ2 = 4.15** ***P*** **= 0.04**
	No	51 (47.7%)	7 (25.9%)	58 (43.3%)	

*P* < 0.05 statistically significant

Among smokeless tobacco (ST) users, 29.2% (*n* = 38) chewed first tobacco within 5 min after waking up in the morning. About 13% (*n* = 17) of ST users intentionally swallowed the tobacco juice. Majority of the female smokeless tobacco users (77.3%, *n* = 34, *P* = 0.001)) felt difficult to quit first tobacco chewed in the morning as compared to males. More than half of the ST users (54.6%, *n* = 71) consumed more than 3 packets/cans of tobacco in a week. Only 17.0% of the ST users chewed more tobacco in the first 1 hour after they woke up than during rest of the day. More than 2/3rd of ST users chewed tobacco even if they were ill and bedridden most of the day (Table 3).

**Table 3:** Percentage of males and females who responded to FTND categories (Smokeless form) visiting outreach program of Dharan, Nepal in June 2018 to April 2019 (*n* = 130)

Questions	Answers	Male (***n*** = 86)	Female (*n* = 44)	Total (***n*** = 130)	***P*** value
1. when do you chew the first tobacco after you wake up?	Within 5 min	22 (25.6%)	16 (36.4%)	38 (29.2%)	**χ2 = 4.52** ***P*** **= 0.21**
	6–30 min	24 (27.9%)	13 (29.5%)	37 (28.5%)	
	31–60 min	12 (14.0%)	8 (18.2%)	20 (15.4%)	
	After 60 min	28 (32.6%)	7 (15.9%)	35 (26.9%)	
2. How often do you intentionally swallow tobacco juice?	Always	12 (14.0%)	5 (11.4%)	17 (13.1%)	**χ2 = 0.38** ***P*** **= 0.82**
	Sometimes	52 (60.5%)	29 (65.9%)	81 (62.3%)	
	Never	22 (25.6%)	10 (22.7%)	32 (24.6%)	
3. which tobacco is most difficult for you to quit?	The first one in the morning	40 (46.5%)	34 (77.3%)	74 (56.9%)	**χ2 = 11.23** ***P*** **= 0.001**
	Any other	46 (53.5%)	10 (22.7%)	56 (43.1%)	
4. How many packets/cans of tobacco do you use per week?	More than 3	52 (60.5%)	19 (43.2%)	71 (54.6%)	**χ2 = 4.04** ***P*** **= 0.13**
	2–3	23 (26.7%)	19 (43.2%)	42 (32.3%)	
	1	11 (12.8%)	6 (13.6%)	17 (13.1%)	
5. Do you chew tobacco more in the first one hour after you wake up than during rest of the day?	Yes	15 (17.4%)	7 (15.9%)	22 (16.9%)	**χ2 = 0.04** ***P*** **= 0.82**
	No	71 (82.6%)	37 (84.1%)	108 (83.1%)	
6. Do you chew tobacco even if you are ill and bedridden most of the day?	Yes	61 (70.9%)	39 (88.6%)	100 (76.9%)	**χ2 = 5.14** ***P*** **= 0.02**
	No	25 (29.1%)	5 (11.4%)	30 (23.1%)	

*P* < 0.05 statistically significant

Nicotine dependence was found in 48% of smokers. About 52% had minimal level of nicotine dependence. Nicotine dependence was found to be higher in females (59%) as compared to males (45%) (Table 4). Nicotine dependence was found in 80% of ST users. More than 90% females had medium or high level of nicotine dependence whereas in males it was found to be 73% (Table 4).

**Table 4:** Classification of FTND score for smoking and smokeless tobacco (ST) users visiting outreach program of Dharan, Nepal in June 2018 to April 2019 (n = 238)

FTND Classification	Male (*n* = 174)		Female (*n* = 64)		Total (***n*** = 238)	
	Smoking (*n* = 107)	ST (*n* = 86)	Smoking (*n* = 27)	ST (*n* = 44)	Smoking (*n* = 134)	ST (*n* = 130)
Minimal (< 4)	59 (55.1%)	23 (26.7%)	11 (40.7%)	3 (6.8%)	70 (52.2%)	26 (20.0%)
Medium (4–6)	39 (36.4%)	36 (41.9%)	13 (48.1%)	24 (54.5%)	52 (38.8%)	60 (46.2%)
High (7–10)	9 (8.4%)	27 (31.4%)	3 (11.1%)	17 (38.6%)	12 (9.0%)	44 (33.8%)

*Note: Total number is greater than 238 as 26 people use both form of tobacco*

Nearly 39% of ST users who initiated tobacco chewing on or before 20 years of age had high level of nicotine dependence as compared to 26.9% who had initiated after 20 years of age. Smokers who had initiated smoking on or before 20 years of age and after 20 years of age had almost same high level of nicotine dependence (8.7% vs 9.5%).

Among the smokeless tobacco users, nicotine dependency was found to be more with increase in duration of tobacco use (AOR = 50.25, 95%CI = 3.51–718.62, *p* = 0.004), low socioeconomic status (AOR = 6.27, 95%CI = 1.30–30.31, *p* = 0.02), less number of tobacco packets used per day and tried to quit tobacco use in last 1 year. Among smokers, nicotine dependency was found to be significantly higher with smoking more than 10 cigarettes per day (AOR = 7.14, 95% CI = 2.00–25.40, *p* = 0.002) (Table 5).

**Table 5:** Classification of FTND score for smoking and smokeless tobacco (ST) users visiting outreach program of Dharan, Nepal in June 2018 to April 2019 (*n* = 238) based on background characteristics

Independent correlate (n)	Univariate OR (95% CI)	Crude *P* value	Adjusted OR (95% CI)	Adjusted *P* value
**Smoking FTND**				
**Sex**				
Male (107)	Ref	–		
Female (27)	1.78 (0.76–4.21)	0.18	0.89 (0.24–3.26)	0.86
**Mean age of initiation of tobacco use**				
≤ 20 (92)	Ref	–		
> 20 (42)	0.88 (0.42–1.82)	0.73	0.76 (0.29–2.03)	
**Total duration of smoking**				
< 10 (66)	Ref	–	–	**–**
10–20 (18)	1.75 (0.61–5.00)	0.29	0.40 (0.09–1.78)	0.23
> 20 (50)	2.85 (1.33–6.10)	0.007	2.24 (0.55–9.10)	0.25
**Socioeconomic status**				
Medium (92)	Ref	–	–	–
Low (42)	1.99 (0.95–4.19)	0.06	2.2 (0.78–6.64)	0.12
**Marital Status**				
Married (85)	Ref	–	–	–
Unmarried/single/ divorced (49)	0.83 (0.34–2.07)	0.70	0.31 (0.07–1.35)	0.12
**Occupation**				
Employed-Skilled (35)	Ref	–	–	–
Employed-Unskilled (42)	0.52 (0.20–1.31)	0.16	0.22 (0.05–0.88)	**0.03**
Unemployed (57)	0.28 (0.11–0.68)	**0.005**	0.29 (0.07–1.18)	0.08
**Alcohol use**				
No (49)	Ref	–	–	–
Yes (85)	1.36 (0.67–2.77)	0.39	2.41 (0.89–6.54)	0.08
**Number of cigarettes smoked/ day**				
Less or equal to 10	Ref	–	–	–
More than 10	6.96 (2.22–21.84)	**0.0009**	7.14 (2.00–25.40)	**0.002**
**Try to quit smoking in last one year**				
Yes	Ref	–		
No	0.93 (0.43–2.04)	0.87	0.57 (0.22–1.43)	0.23
**Family member smoking**				
No	Ref	–	–	–
Yes	1.11 (0.56–2.19)	0.76	1.11 (0.45–2.71)	0.81
**Smokeless Tobacco FTND**				
**Sex**				
Male (86)	Ref	–	–	–
Female (44)	4.98 (1.41–17.69)	**0.01**	2.239 (0.36–13.57)	0.38
**Mean age of initiation of tobacco use**				
≤ 20 (78)	Ref	–		
> 20 (52)	1.13 (0.47–2.69)	0.78	2.12 (0.39–11.39)	0.37
**Total duration of tobacco use**				
< 10 (47)	Ref	–	–	–
10–20 (32)	5.18 (1.57–17.16)	**0.007**	4.58 (0.78–26.88)	0.09
> 20 (51)	18.14 (3.93–83.60)	**0.000**	50.25 (3.51–718.62)	**0.004**
**Socioeconomic status**				
Medium (77)	Ref	–	–	–
Low (53)	2.75 (1.02–7.40)	**0.04**	6.27 (1.30–30.31)	**0.02**
**Marital Status**				
Married (109)	Ref	–	–	–
Unmarried/ single/ divorced (21)	0.14 (0.05–0.40)	**0.0002**	0.77 (0.08–6.97)	0.82
**Occupation**				
Employed-Skilled (32)	Ref	–	–	–
Employed-Unskilled (51)	2.50 (0.77–8.04)	0.12	0.76 (0.13–4.23)	0.75
Unemployed (47)	0.97 (0.34–2.73)	0.95	1.04 (0.17–6.19)	0.96
**Alcohol use**				
No (71)	Ref	–	–	–
Yes (59)	0.31 (0.13–0.75)	**0.01**	0.47 (0.11–1.95)	0.30
**Number of packets/cans of smokeless tobacco chewed/day**				
Less or equal to 1	Ref	**–**	**–**	**–**
More than 1	0.44 (0.18–1.06)	0.068	0.07 (0.01–0.32)	**0.001**
**Try to quit tobacco use in last one year**				
Yes	Ref	**–**	**–**	**–**
No	0.57 (0.18–1.82)	0.93	0.18 (0.03–0.96)	**0.04**
**Family member chewing tobacco**				
No	Ref	**–**	**–**	**–**
Yes	1.03 (0.43–2.46)	0.93	0.83 (0.21–3.21)	0.79

Univariate and binary logistic regression analyses were used

CI Confidence Interval

OR odds ratio

*P* < 0.05 statistically significant

## Discussion

Various studies had been conducted in Nepal regarding the prevalence of tobacco use (smoking and smokeless form). But there is dearth of studies which have assessed the level of nicotine dependence. This study was done to assess the level of nicotine dependence among smokers and smokeless tobacco users in outreach program of BPKIHS-Dharan, Nepal. Therefore, this study would add data in the level of nicotine dependence among smoking and smokeless tobacco users in Nepal.

Proportion of smoking and smokeless tobacco users were not comparable to population surveys. Therefore, no comparison was made regarding prevalence of tobacco use. Almost 45% of tobacco users had positive family history of tobacco use. It has been shown that habit of tobacco use runs in families [16]. More than half of the tobacco users (127, 53.4%) also consumed alcohol. Alcohol consumption was found to be more among males (102, 58.6%) than females (25, 39.1%). Study also confirmed the co-use of tobacco and alcohol [17]. Mean age of initiation of tobacco smoking was found to be almost similar (19.10 ± 5.939) to STEPS survey (18.2 years) [18].

About one in three (almost 29%) tobacco users used the first tobacco immediately or within 5 minutes of waking up which is in contrast to GATS: India 2016–17 Report [19] where about one in five (18%) daily tobacco users resorted to tobacco use immediately or within 5 minutes. This indicates that dependency of tobacco was high in our study population.

Almost 48% of smokers had medium and high level of nicotine dependency which is almost similar to the study done by Aryal et al. [13] where about 51% had medium and high level of nicotine dependency. However high level of nicotine dependency was found in only 9% as compared to 20.4% in Aryal et al. [13]. This study was conducted exclusively in a community level which comprises individuals from both rural and urban settings.

Till date no any study has been published in Nepal regarding nicotine assessment of smokeless tobacco users. In our study nicotine dependence was seen more in smokeless tobacco users (80%) as compared to smokers (48%). Higher median duration of use of ST in comparison to smoking (17 years versus 11.5 years) may be one of the factors. This could also be due to high nicotine content in smokeless tobacco as compared to cigarette smoking [20].

Only 24.24% (64) tried to quit tobacco at least 1 or 2 times in last 1 year. More percentage of male tobacco users (51, 26.4%) tried to quit in comparison to female (13, 18.3%). Evidence suggests that women were significantly less likely to quit smoking than men both due to biological and psychological factors, suggesting that the addictiveness of smoking may be greater for females [21].

The various factors for high level of nicotine dependency include: lower income, lower education, younger age of first smoking [22], high alcohol dependence [22, 23], mood and anxiety disorders [24] and genetic factors [22, 25]. High nicotine dependence is associated with lower quality of life, lower work productivity and higher health-care use [26]. Lower level of education, more duration of tobacco use, history of less previous quit attempt in last 1 year and elder females may be the factors associated with more nicotine dependency level seen in female in our study. Most of the previous studies were done considering only smoking form of tobacco whereas our study considered both forms of tobacco i.e. smoking and ST.

### Strengths and limitations of the study

First, social desirability bias [27] might have occurred. As the tobacco use status was obtained through self-report without biochemical verification, which might lead to reporting inaccuracy. There may be a possibility of information bias especially related to history of tobacco use and number of cigarettes/tobacco can or pouches used per day. Additionally, number of cigarettes, packs of smokeless tobacco may have been under-reported leading underestimation of level of dependence.

Second, the study was based on non-probability sampling (convenience sampling). The effect of outliers can be seen in this kind of subject selection. Outliers are cases who are considered as not belonging to the data [28]. Therefore, the result shows associations but do not deliver evidence for causality.

Third, this study might hide the true prevalence as smoking by women is socially unacceptable. The cultural and geographical variation might affect tobacco use and level of nicotine dependence [29].

Fourth, face and content validity of the questionnaire was done but construct validity of the questionnaire was not done which is also the limitation of the study. Further studies should be conducted to validate Nepalese version of FTND.

Fifth, AOR confidence intervals were wide, hence, the study lacked statistical power.

Although the convenience sampling was used, our study covered more than 30% of the districts of province number one. The data collection was done in various parts of the selected districts. Moreover, the results obtained from this study can be generalized in province number one. Further studies are required to confirm the dependency using the bio-markers. A large nationwide study should be done to assess the level of nicotine dependence as this study shows the high level of nicotine dependency among smokeless tobacco users as well as smokers.

### Recommendations

As nicotine dependency is high in our study population, it is very important to develop effective tobacco cessation program. The government of Nepal should provide effective health education (HE) to raise the awareness and motivation to quit tobacco. Along with HE, nicotine replacement therapy (NRT) should start from government level to reduce the level of nicotine dependence and tobacco use prevalence. As this study provides insight about high level of nicotine dependency among smokeless tobacco users the government of Nepal should focus on reducing not only of smoking prevalence but also smokeless tobacco use.

## Conclusions

Our study concluded that nicotine dependence (Moderate and severe) was more prevalent in ST users (80%) in comparison to smokers (48%).

## Data Availability

The datasets analyzed during the current study and questionnaires in Nepali language that were used in this study are available from the corresponding author on reasonable request.

## Appendix 1

### Original version of Fagerström Test for Nicotine Dependence

1. How soon after you wake up do you smoke your first cigarette?

- Within 5 min
- 6 to 30 min
- 31 to 60 min
- After 60 min

2. Do you find it difficult to refrain from smoking in places where it is forbidden (e.g., in church, at the library, in the cinema)?

- No
- Yes

3. Which cigarette would you hate most to give up?

- The first one in the morning
- Any other

4. How many cigarettes per day do you smoke?

- 10 or less
- 11 to 20
- 21 to 30
- 31 or more

5. Do you smoke more frequently during the first hours after waking than during the rest of the day?

- No
- Yes

6. Do you smoke when you are so ill that you are in bed most of the day?

- No
- Yes

### Modified Fagerström Test for Nicotine Dependence for smokers used in our study

**Modified Fagerström Test for Nicotine Dependence for smokers used in our study:**

1. When do you smoke your first cigarette after waking up?

a. Within 5 min ......... 3 b. 6–30 min ............. 2.

c. 31–60 min .......... 1 d. After 60 min......... 0.

2. Do you feel difficult when you are not allowed to smoke in the smoking restricted areas (like temple, library, Cinema hall etc.)

a. Yes...... 1 b. No ..... 0.

3. Which cigarette is most difficult for you to quit?

a. The first one in the morning ........ 1 b.Any other ....... 0.

4. How many cigarettes do you smoke per day?

a. 10 or less ....... 0 b. 11–20........ 1.

c. 21–30...... 2 d. 31 or more ...... 3.

5. Do you smoke more frequently during the first hours after waking than during the rest of the day?

a. Yes...... 1 b. No .... 0.

6. Do you smoke even if you are ill and bedridden most of the day?

a. Yes...... 1 b. No ...... 0

### Original version Fagerström Test for Nicotine Dependence for smokeless tobacco users

1. How soon after you wake up to do you place your first dip?

- Within 5 min….. 3
- 6–30 min….. 2
- 31–60….. min 1
- After 60 min….. 0

2. How often do you intentionally swallow tobacco juice?

- Always….. 2
- Sometimes….. 1
- Never….. 0

3. Which chew would you hate to give up most?

- The first one in the morning….. 1
- Any other….. 0

4. How many cans/pouches per week do you use?

- More than 3….. 2
- 2–3….. 1
- 1….. 0

5. Do you chew more frequently during the first hours after awakening than during the rest of the day?

- Yes….. 1
- No….. 0

6. Do you chew if you are so ill that you are in bed most of the day?

- Yes….. 1
- No….. 0

### Modified Fagerström Test for Nicotine Dependence for smokeless tobacco users used in our study

1. When do you chew the first tobacco after you wake up?

a. Within 5 min ............ 3 b. 6–30 min ........ 2.

c. 31–60 min ......... 1 d. After 60 min ........ 0.

2. How often do you intentionally swallow tobacco juice?

a. Always ......... 2 b. Sometimes ....... 1 c. Never ...... 0.

3. which tobacco is most difficult for you to quit?

a. The first one in the morning ......... 1 b. Any other ......... 0.

4. How many packets/cans of tobacco do you use per week?

a. More than 3 .......... 2 b. 2–3 ....... 1 c. 1 ...... 0.

5. Do you chew tobacco more in the first one hour after you wake up than during rest of the day?

a. Yes ........ 1 b. No ....... 0.

6. Do you chew tobacco even if you are ill and bedridden most of the day?

a. Yes ...... 1 b. No ...... 0.

## Appendix 2

**Table 6:** Tobacco prevalence among people visiting outreach program of Dharan, Nepal in June 2018 to April 2019 (*n* = 726)

Sex	Total number of people surveyed	Smoking Prevalence	Smokeless Tobacco Prevalence	Both form of Tobacco use Prevalence	Overall Tobacco use prevalence
**Male**	406	20.9% (88)	16.5% (67)	4.7% (19)	174 (42.8%)
**Female**	320	6.2% (20)	11.6% (37)	2.2% (7)	64 (20.0%)
**Total**	726	14.9% (108)	14.3% (104)	3.6% (26)	238 (32.8%)

## Abbreviations

AOR: Adjusted odds ratio
BPKIHS: B.P. Koirala Institute of Health Sciences
CI: Confidence interval
CODS: College of Dental surgery
FTND: Fagerstrom Test for Nicotine Dependence
GATS: Global Adult Tobacco Survey
HE: Health Education
ICD: International Classification of Disease
LICs: Low-income countries
NRT: Nicotine replacement therapy
OR: Odds ratio
SEA: South-East Asia
SES: Socioeconomic status
SPSS: Statistical Package for Social Sciences
ST: Smokeless tobacco
